# The comprehensive study on the role of POSTN in fetal congenital heart disease and clinical applications

**DOI:** 10.1186/s12967-023-04529-1

**Published:** 2023-12-11

**Authors:** Yi Xia, Liang Chen, JinWen Lu, Jianhong Ma, Yuanzhen Zhang

**Affiliations:** 1 Department of Obstetrics, Women’s and Children’s Hospital, Zhongnan Hospital of Wuhan University, 169 Donghu Road, Wuchang District, Wuhan, 430071 Hubei China; 2 Department of Ultrasound, Wuhan University Zhongnan Hospital, Wuhan, 430071 Hubei China; 3 Clinical Research Center for Prenatal Diagnosis and Birth Health of Hubei Province, Wuhan, Hubei China; 4 Clinical Research Center for Reproductive Science and Birth Health of Wuhan, Wuhan, Hubei China

**Keywords:** Congenital heart disease, Screening, POSTN, PAPPA, Marker

## Abstract

**Background:**

Congenital heart defect (CHD) is the most common congenital abnormality, and it has long been a clinical and public health concern. Our previous findings have found Periostin (POSTN) and Pappalysin-1 (PAPPA) as potential biomarkers for fetal CHD. We aim to further elucidate POSTN's role in fetal heart development and explore the clinical applicability of POSTN and PAPPA as diagnostic marker for fetal CHD. This study is poised to establish a theoretical framework for mitigating the incidence of CHD and advance a novel approach for prenatal screening of fetal CHD.

**Methods:**

We verified differential expression of POSTN and PAPPA in gravida serum and fetal amniotic fluid based on our previous research. We established the *Postn* knockout mouse by CRISPR/Cas9 to investigate whether *Postn* deletion leads to cardiac abnormalities in mice. Besides, we explored the mechanism of POSTN on heart development through *Postn* knockout mouse model and cell experiments. Finally, we established the logistic regression model and decision curve analysis to evaluate the clinical utility of POSTN and PAPPA in fetal CHD.

**Results:**

We observed a significant decrease in POSTN and increase in PAPPA in the CHD group. Atrial septal defects occurred in *Postn*^*−/−*^ and *Postn*^±^ C57BL/6 fetal heart, while ventricular septal defects with aortic saddle were observed in *Postn*^±^ C57BL/6 fetal heart. Disruption of the extracellular matrix (ECM) in cardiomyocytes and multiple abnormalities in cellular sub-organelles were observed in *Postn* knockout mice. POSTN may positively regulate cell behaviors and unsettle ECM via the TGFβ-Smad2/3 signaling pathway. The combination of serum biomarkers POSTN and PAPPA with Echocardiogram can enhance the diagnostic accuracy of CHD. Furthermore, the comprehensive model including POSTN, PAPPA, and two clinical indicators (NT and age) exhibits significantly higher predictive ability than the diagnosis group without the use of serum biomarkers or clinical indicators.

**Conclusions:**

It is the first evidence that *Postn* deletion leads to cardiac developmental abnormalities in fetal mice. This may involve the regulation of the TGFβ signaling pathway. Importantly, POSTN and PAPPA possess clinical utility as noninvasive prenatal promising screening indicators of CHD.

**Supplementary Information:**

The online version contains supplementary material available at 10.1186/s12967-023-04529-1.

## Introduction

Congenital heart disease (CHD) primarily refers to structural or functional abnormalities of the heart and its vessels during embryonic development [1]. With an incidence rate of approximately 2.3/1000–9.1/1000, CHD is the most common birth defect in China, Europe [2], the United States [3], and many other countries worldwide [4], accounting for one-third of all major congenital anomalies [4]. CHD affects approximately 0.8% of live births [5], and 15% of these cases result in perinatal death due to severe malformations, making it a major cause of infant mortality and disability.

Currently, the pathogenesis of CHD is primarily associated with abnormal extracellular matrix (ECM), restricted migration of mesenchymal cells, cell necrosis, and intracardiac hemodynamic abnormalities [1]. Our previous investigations have suggested that the ECM protein periostin (POSTN) could serve as a potential marker for fetal CHD, with the ECM playing a crucial role as a medium for cell activity and signal transmission. Notably, the TGF-β signaling pathway is a crucial signaling pathway for ECM [6]. Therefore, we aim to explore the potential relationship between the changes in POSTN expression and cellular behavior during CHD development, as well as the potential role of POSTN in the TGF-β signaling pathway.

In the realm of diagnosing fetal CHD, the primary modality employed is echocardiography (ECG). Despite its widespread use, this diagnostic method has demonstrated a notable rate of missed diagnoses, which can range anywhere from 30% [7] to 50% [8]. Furthermore, ultrasound diagnosis of fetal CHD is typically conducted during the second and third trimesters, with pregnancy termination during this period potentially causing significant physical and mental harm to the pregnant women. Therefore, the identification of objective markers for CHD is essential to improve early diagnosis. The ideal approach for prenatal diagnosis of fetal diseases involves identifying fetal disease markers in the peripheral blood of pregnant women. This noninvasive method is handy and can eliminate the risks associated with invasive prenatal diagnosis(amniocentesis), making it easily accepted by patients. Our previous research has uncovered an interaction between POSTN and Pappalysin-1 (PAPPA), both of which are potential biomarkers for CHD. Therefore, we aim to further evaluate the clinical value of POSTN and PAPPA in the diagnosis of fetal CHD based on previous resulets.

## Methods

### Enzyme linked immunosorbent assay, ELISA

Gravida serum (GS) and amniotic fluid (AF) samples were analyzed for POSTN (A102870-96 T, Shanghai Fu Sheng Industrial Co., Ltd., China) and PAPPA (HM11110, Bioswap, China) using commercial ELISA kits, according to the manufacturer’s instructions.

### The breeding, sampling and genetic identification of C57BL/6

*Postn*^±^ C57BL/6 aged 5–8 weeks were purchased from Saiye Inc. (Suzhou, China) and housed following the 3R principle at the Animal Experiment Center of Zhongnan Hospital of Wuhan University. The appearance of a mating plug was considered embryonic day 0.5 (E0.5). At embryonic day 15 (E15), embryonic day 20 (E20), and the day of birth, the hearts were extracted from the mice and subjected to visual inspection under a stereomicroscope for quality control purposes. This inspection encompassed an assessment of the integrity of crucial structures, including the heart contour, ventricles, atria, and aortic structures. Hearts that met quality control criteria were fixed with 4% paraformaldehyde for subsequent experiments. DNA was extracted from tail and yolk sac samples of mice using an alkaline lysis method and analyzed through 2% agarose gel electrophoresis for genetic identification. The specific information regarding primers, PCR reaction conditions, and reagents used in this part is provided in Additional file 1. This study was approved by the Ethics Committee of the Animal Experiment Center of Zhongnan Hospital of Wuhan University (ethical approval number: ZN2021236).

### The collection of clinical samples

This study enrolled 160 pregnant women who visited the Department of Obstetrics and Gynecology at Zhongnan Hospital of Wuhan University between October 2020 and December 2022. Of these, 80 pregnant women in the CHD group had fetuses diagnosed with CHD by echocardiography, while the other 80 subjects in the control group with normal results of prenatal diagnosis and echocardiography during the same period. Diagnostic criteria for CHD are based on expert consensus on the diagnosis of fetal CHD and perinatal management [9]. The study was approved by the Ethics Committee of Zhongnan Hospital of Wuhan University (Project Number: 2020085) and conducted according to the Declaration of Helsinki. Informed consent was obtained from every patient.

### The paraffin-embedding technique, hematoxylin–eosin staining (HE), and Masson staining

The mouse heart tissue sections, which were obtained from paraffin-embedded samples, were continuous and measured 5 μm in thickness. these sections were HE staining and Masson staining. Methods for paraffin embedding, sectioning, HE staining and Masson staining and are available at the website (https://www.servicebio.cn/data-center?code=SYCZSP). Subsequently, these stained tissue sections were scanned using an Aperio ePathology Scanner (Leica, Germany) and underwent statistical analysis using the ImageJ software (NIH, USA).

### Real-time quantitative PCR (RT-qPCR)

The process of RT-qPCR involved total RNA extraction and identification, reverse transcription to cDNA, and other processes with the same detailed process as previously described [11]. The expression of target genes was calculated using the 2^−△△Ct^. Details of the primers and procedures in this experiment are provided in Additional file 1.

### Western blotting (WB)

WB involves protein extraction, concentration measurement, electrophoresis, transmembrane, antibody incubation, chemiluminescence detection, and quantitative analysis. Experimental details are available at the website (https://www.servicebio.cn/data-center?code=WBSYLC). Antibodies used in this study are listed in Additional file 1.

### Transmission electron microscope (TEM)

Mouse myocardial tissue samples of approximately 1 mm^3^ were fixed in pre-chilled 2.5% glutaraldehyde for two weeks within 1 min of excision. For further details refer to the website (https://www.servicebio.cn/data-detail?id=4304&code=DJSYBG). Ultrathin sections were analyzed and imaged using a Hitachi HT7700 transmission electron microscope.

### Cell culture, knockdown and overexpression of *Postn*

H9c2 cells were obtained from the Department of Cardiology, Zhongnan Hospital of Wuhan University, rat cardiac fibroblasts (RCF) were purchased from Beina Biological, and P19 cells were purchased from Punosai Life Science Co., Ltd. These cells were cultured at 37 °C with 95% air and 5% CO2. After transient transfection of siRNA (plvx-shRNA2-ZSGreen-T2A-puro, Hippo Biotechnology Co., Ltd., China) to identify the most efficient siRNA for *Postn* knockdown, cell lines with knockdown *Postn* and Overexpressing *Postn* (pLV6ltr-ZsGreen-Puro-CMV). Sequences of reagents, and siRNAs used in this experiment are provided in Additional file 1.

### Adhesion, migration, invasion, and differentiation of cells

The specific steps of cell adhesion, migration, transwell invasion and differentiation of P19 cells into cardiomyocytes are shown in Additional file 2.

### Statistical analysis

Data analysis was performed using SPSS 22.0 (SPSS Inc, Chicago, IL) and figures were produced with GraphPad Prism9 (GraphPad, La Jolla, USA). Continuous data were reported as Mean ± SD and categorical variables as percentages. Two-group comparisons of continuous variables were performed using *t*-tests when normality assumptions were met, while one-way ANOVA was employed for multiple group comparisons. Nonparametric tests were used when normality assumptions were violated. Chi-square tests were used to analyze categorical variables. *P*-value < 0.05 was considered statistically significant.

## Results

### The basic characteristics of the subjects

Table 1 presents the subjects’basic characteristics. No significant differences were observed between the groups in maternal and male age, fetal nuchal translucency (NT), and noninvasive prenatal genetic testing (NIPT)/Down screening results.

**Table 1:** Basic characteristics of the subject

	CHD group	Control group	*P* value
Elisa validation sets (n = 160)	80	80	
Maternal age (Year)	30.63 ± 5.3	31.86 ± 6.73	0.897
Gestational age (week)	22.664 ± 2.637	21.116 ± 2.601	0.002
NT (cm)	0.152 ± 0.071	0.172 ± 0.069	0.072
NIPT/Down's screening			0.741
Low-risk	79	67	–
High-risk	1	13	–

NT, fetal nuchal transparent zone; NIPT, non-invasive prenatal genetic testing. *P* < 0.05 showed statistical differences

### Significant decrease of POSTN and increase of PAPPA in GS and AF

**Fig. 1:**
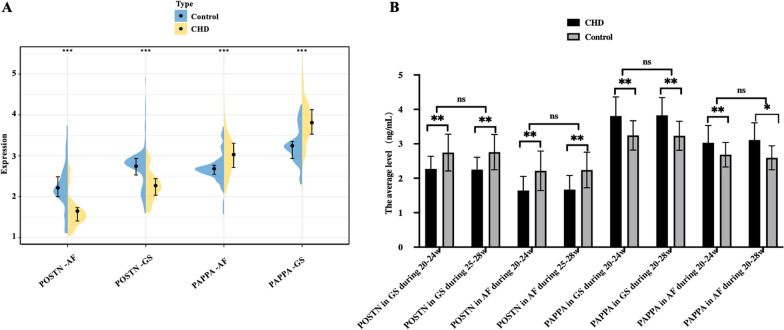
The expression of POSTN and PAPPA in GS and AF. GS, gravida serum; AF, amniotic fluid; CHD, congenital heart disease. **A** the expression of POSTN and PAPPA in GS and AF in this research. **B** the expression of POSTN and PAPPA in AF and GS did not exhibit a significant difference between the 20–24 weeks and 25–28 weeks of pregnancy (*P* > 0.05). However, consistent with the findings depicted in Fig. 1A, it was observed that irrespective of the 20–24 or 25–28 week interval, the CHD group exhibited significantly lower levels of POSTN compared to the control group (*P* < 0.05), while PAPPA levels were significantly higher in the CHD group compared to the control group. *P < 0.05,**P < 0.01,***P < 0.001

To corroborate our previous findings, we conducted ELISA on GS and AF to assess POSTN and PAPPA levels before embarking on mechanistic and clinical study. As depicted in Fig. 1A, compared to the control group, the CHD group exhibited lower POSTN concentrations in both GS and AF (2.27 ± 0.368 ng/mL vs 2.746 ± 0.564 ng/mL, *P* < 0.001; 1.646 ± 0.409 vs 2.217 ± 0.574 ng/mL, *P* < 0.001, respectively). Conversely, PAPPA levels in GS and AF were higher in the CHD group compared to the control group (3.81 ± 0.554 ng/ml vs 3.245 ± 0.427 ng/ml, *P* < 0.005; 3.029 ± 0.505 vs 2.684 ± 0.356 ng/ml, *P* < 0.05). As illustrated in Fig. 1B, the expression of POSTN and PAPPA in amniotic fluid (AF) and gestational sac (GS) did not exhibit a significant difference between the 20–24 weeks and 25–28 weeks of pregnancy (*P* > 0.05). Furthermore, in line with the result of Fig. 1A, the CHD group displayed a significantly lower level of POSTN compared to the control group (*P* < 0.05), whereas PAPPA was significantly higher in the CHD group compared to the control group.

### *Postn* knockout mice fetuses develop structural cardiac malformations

**Fig. 2:**
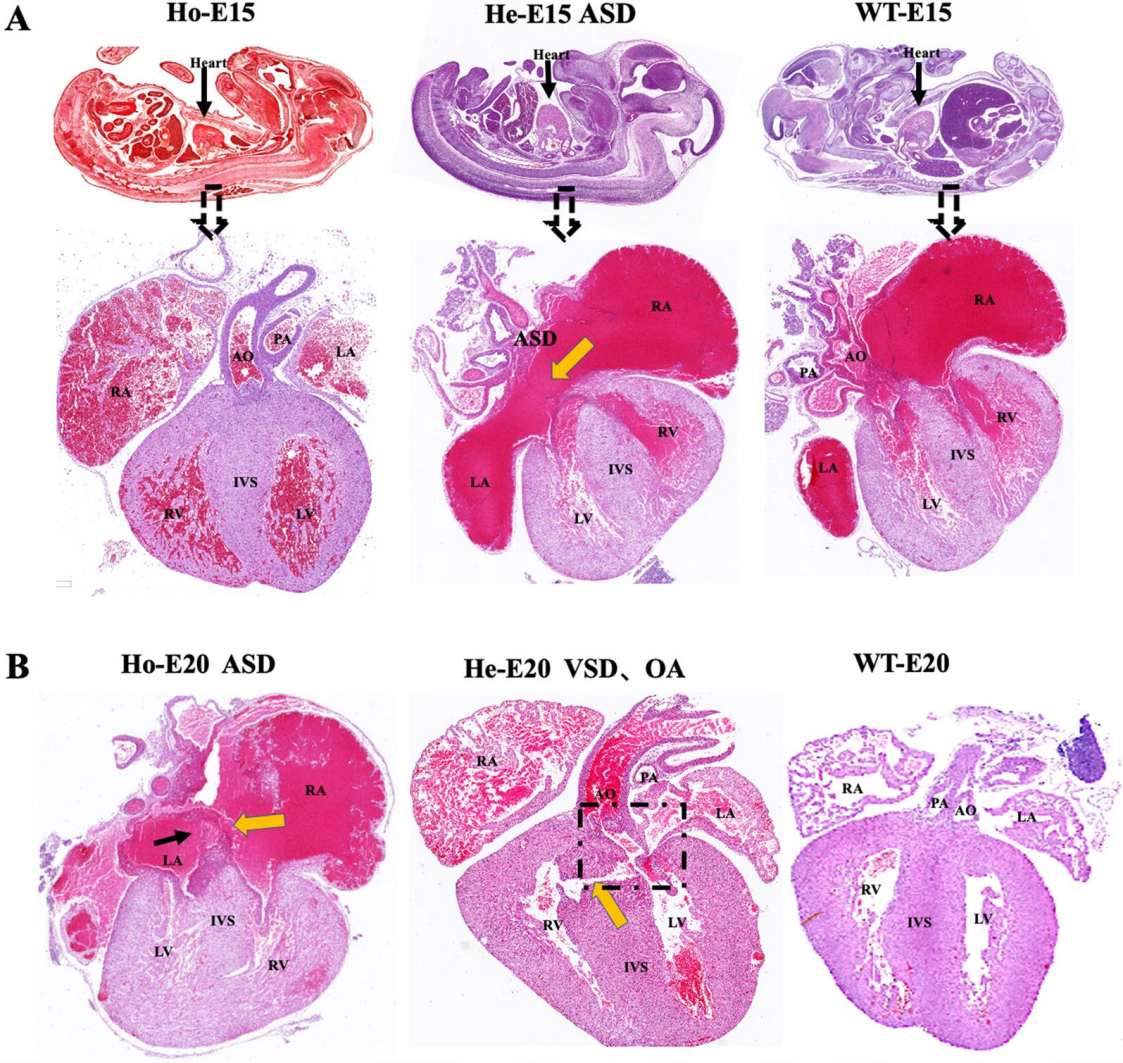
Coronal and sagittal views of the heart at E15 and E20. Ho, homozygous; He, heterozygous; WT, wild type; RA, right atrium; LA, left atrium; PA, pulmonary artery; IVS, interventricular septum; RV, right ventricle; LV, left ventricle; ASD, atrial septal defect; AO, aorta; E15, the embryo on the 15th day of embryonic period; E20, the embryo on the 20th day of embryonic period. **A** the He-E15 developed ASD (shown by yellow arrows).** B** Ho-E20 developed ASD (indicated by black arrows) combined with right atrial diverticulum (RAD, indicated by yellow pointed tips); and He-E20 developed VSD (indicated by yellow arrows) and aortic saddle (the ventricles were connected to the aorta and pulmonary artery, the left ventricle was connected to the aorta, and the aorta saddled above the ventricular septal defect, as the dashed box shown)

*Postn* knockout in C57BL/6 fetuses resulted in the heart structural defects. Heterozygous (He) fetuses at embryonic day 15 (He-E15) displayed large atrial septal defects, while homozygous (Ho) fetuses at embryonic day 20 (Ho-E20) presented with atrial septal defects and atrial diverticulum. Additionally, heterozygous (He) fetuses at embryonic day 20 (He-E20) exhibited ventricular septal defects combined with aortic saddle, as displayed in Fig. 2.

### No discernible abnormalities in cardiac function were observed in *Postn* knockout C57BL/6 mice

**Fig. 3:**
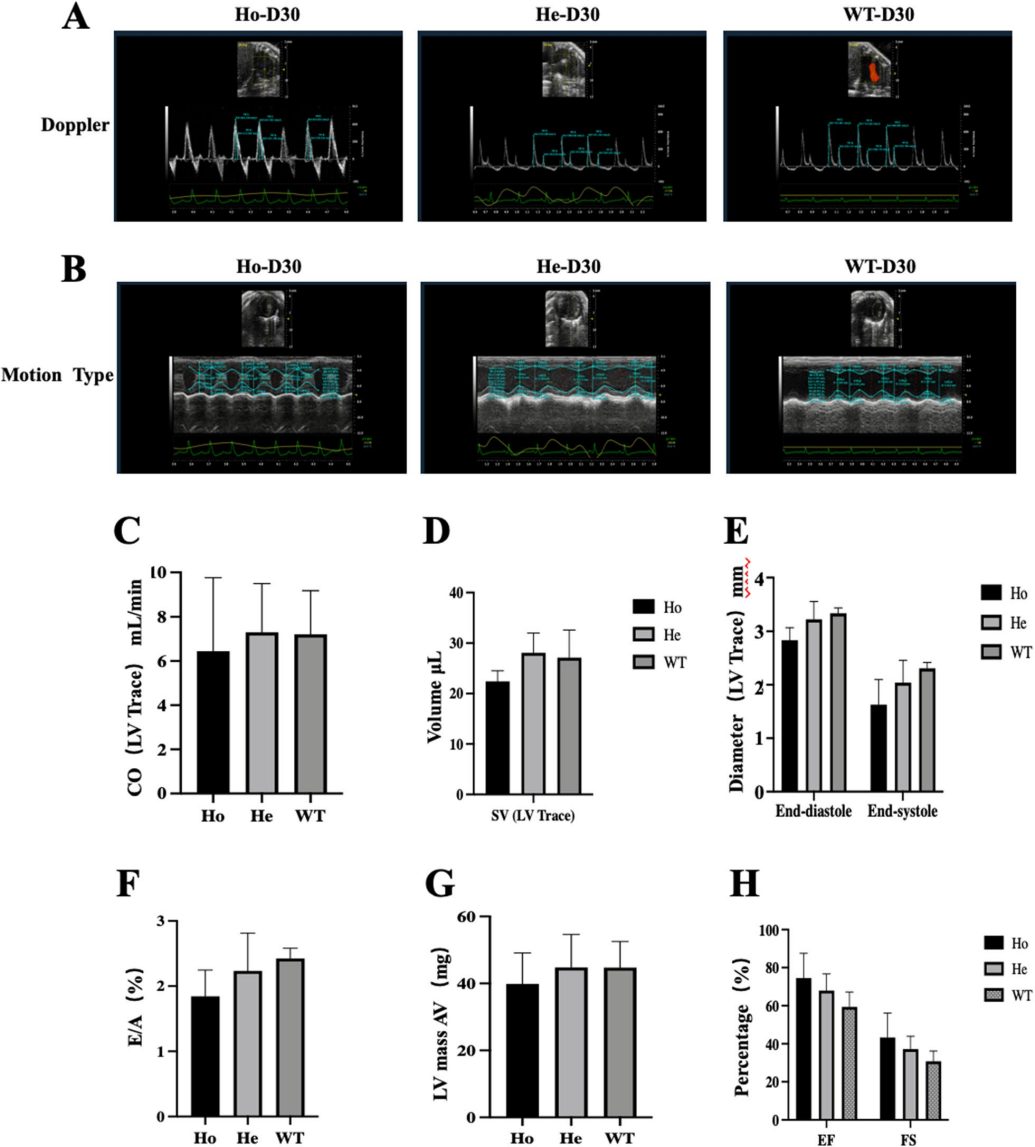
The echocardiographic assessment of the mice heart on D30. Ho, homozygous(n = 5); He, heterozygous(n = 5); WT, wild type(n = 5); LV, left ventricle; CO, cardiac output; SV, stroke volume; E/A, the ratio of the peak values of blood flow velocity through the mitral valve in the early and late diastolic period. EF, left ventricular ejection fraction; FS, fraction shortening of short-axis. **A** and **B** are Doppler sonograms and M-mode sonograms of mouse hearts, respectively. **C** represents the cardiac output (LV trace). **D** shows the ventricular stroke volume. **E** refers to the left ventricular diameter at the end of systole and diastole. **F** shows the ratio of the peak values of blood flow velocity through the mitral valve in the early and late diastolic period. **G** represents that maximum thickness of the anterior wall (AW) of the left ventricle. The above statistical analysis results showed no significant differences(*P* > 0.05). **H** EF represents the ejection fraction. FS represents the fractional shortening of the left ventricle

Cardiac dysfunction is a crucial aspect of CHD. In order to investigate whether *Postn* knockout mice exhibit any abnormalities in cardiac function, we performed echocardiography on 30-day-old mice. As illustrated in Fig. 3, there were no statistically significant differences in cardiac output (CO), stroke volume (SV), left ventricular end-systolic and end-diastolic volumes (Diameter; s/d), E/A values, maximum left ventricular anterior wall thickness (LV Mass AW, Corrected), left ventricular ejection fraction (EF), and fractional shortening (FS) among the three groups of Ho, He, and WT (*P* > 0.05). However, these results cannot definitively establish the absence of abnormal cardiac function in *Postn* knockout mice.

### *Postn* knockout mice with ECM disturbances

**Fig. 4:**
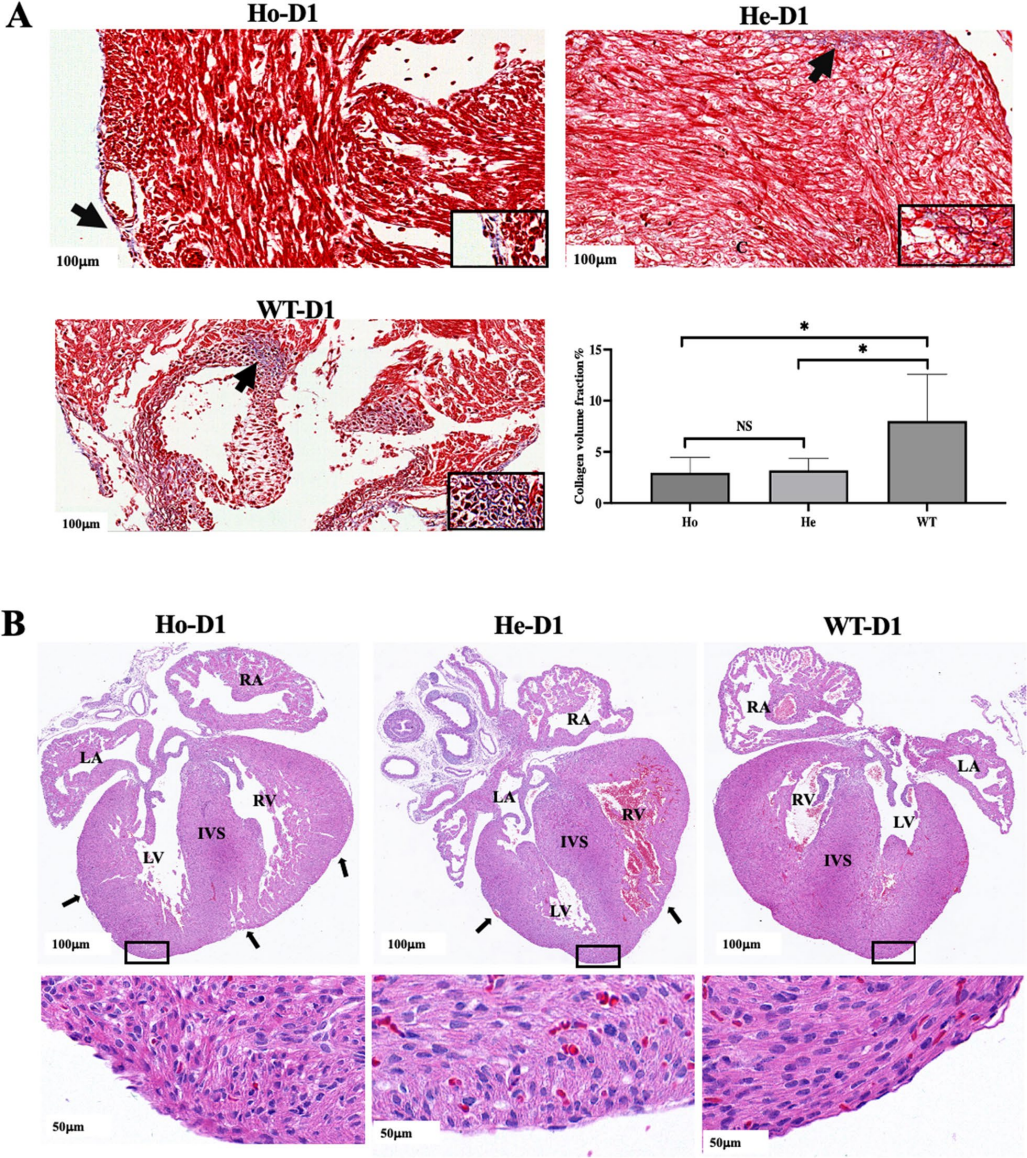
ECM abnormalities in the mice heart on the day of birth(D1). Ho, homozygous(n = 10); He, heterozygous(n = 10); WT, wild type(n = 10); RA, right atrium; LA, left atrium; AO, aorta; PA, pulmonary artery; IVS, ventricular septal; RV, right ventricle; LV, left ventricle; CVF, collagen volume fraction. Figure **A** shows that the CVF of Ho and He were significantly reduced and there was less collagen in the ECM compared to WT. As shown in Figure** B** on the coronal plane of the heart, the muscular wall space/trabecular meshwork capsule was obvious in Ho mice D1. For He mice, large small capsules between muscle walls were obviously visible in the heart. Enlarged view of the apex of the heart in the mouse shows that, Ho and He mice myocardial cells are arranged disorderly, the intercellular space is enlarged, and the proportion of cells with irregularly shaped nuclei increased

To investigate the impact of *Postn* knockout on heart ECM, we performed Masson and HE staining of mice hearts at birth day (D1). As illustrated in Fig. 4A, results from Masson staining revealed a significant reduction in collagen volume fraction (CVF) in the hearts of Ho mice when compared to WT mice. While, no significant difference in CVF was observed between He and WT mice. In contrast to WT mice, Fig. 4B demonstrated that Ho mice exhibited widened intercellular gaps, disorganized cells, irregular nuclear morphology, enlarged space within the muscular wall, and evident pouches in myocardial trabeculae within the heart. And slightly loose intercellular spaces and increased number of irregular nuclei were observed in the He mice heart. These findings suggest that genetic deletion of the Postn gene in mice results in disruption of the heart ECM.

### Ultrastructural abnormalities of cardiac organelles in *Postn* knockdown mice

**Fig. 5:**
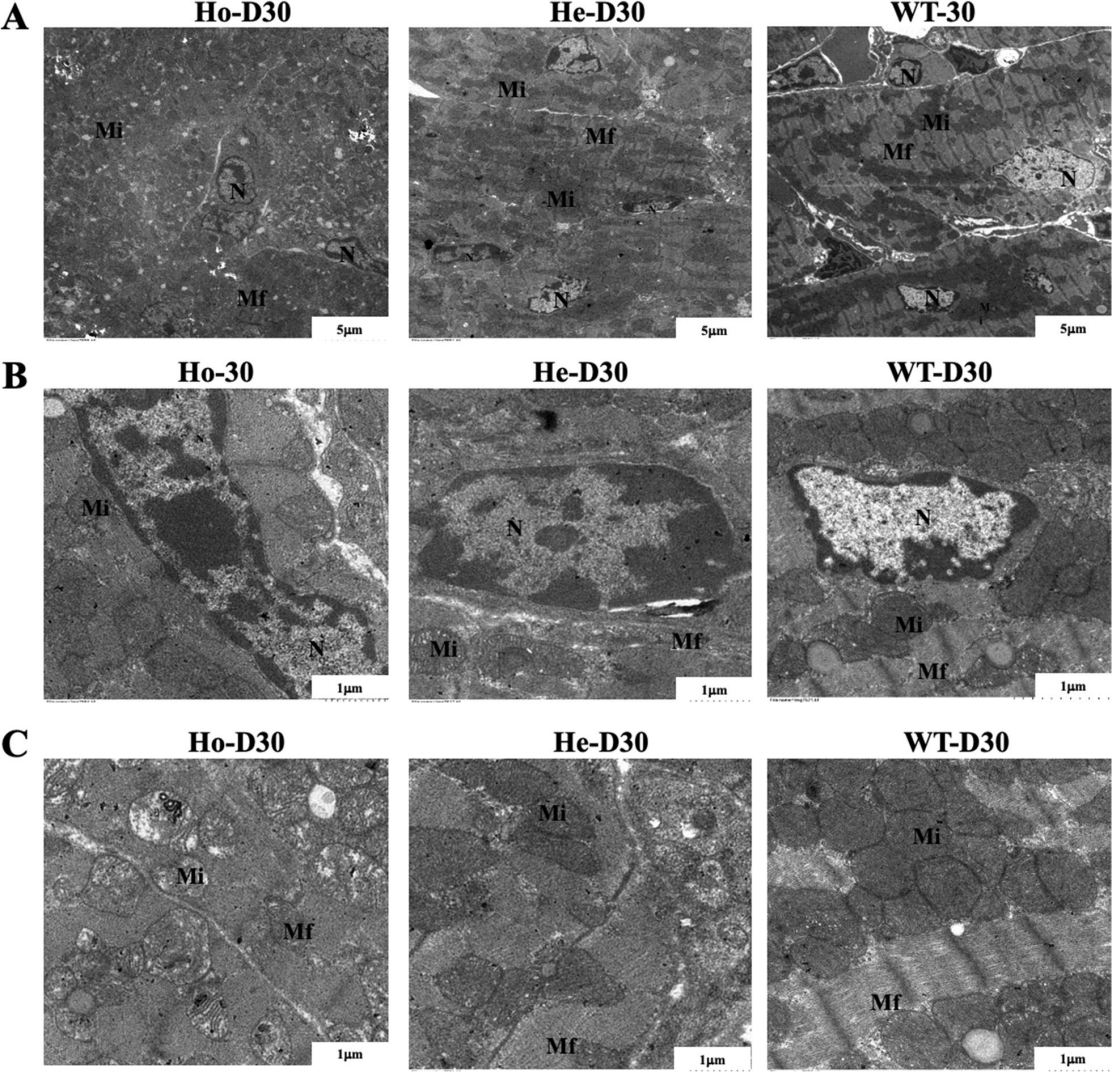
Transmission electron microscopic view of the mice heart on D30. Ho, homozygous(n = 10); He, heterozygous(n = 10); WT, wild type(n = 10); Mf, myofibril; Mi, mitochondria; N, nucleus. It can be seen from the figure that the field of view of Ho was confused (**A**), no clear Mf structure was observed, the intracellular organelles were blurred, the nucleus (**B**) was irregular in shape, the nuclear membrane was discontinuous and smooth, the nucleoli were enlarged, and euchromatin was reduced; mitochondria (**C**) were significantly proliferated and abnormal; The He sample showed that Mf was less neatly arranged and heterochromatin was increased (**B**)

We employed transmission electron microscopy to explore whether cardiomyocyte ultrastructure is affected by *Postn* deletion. As illustrated in Fig. 5, In Fig. 5A, Ho samples exhibited a visually confused field, with blurred and irregularly arranged myofibrils, disorganized intracellular organelles, and irregularly shaped nuclei (Fig. 5B) characterized by discontinuously smooth nuclear membranes, enlarged nucleoli, and reduced euchromatin. Furthermore, mitochondria (Fig. 5C) were significantly proliferated, with numerous vacuoles and floccules observed. The He sample displayed less orderly arranged myofibrils and reduced euchromatin. These findings suggest that *Postn* knockout results in abnormal morphology and structure of multiple suborgans in mice myocardial cells.

### POSTN can positively regulate cell adhesion, migration and invasion

**Fig. 6:**
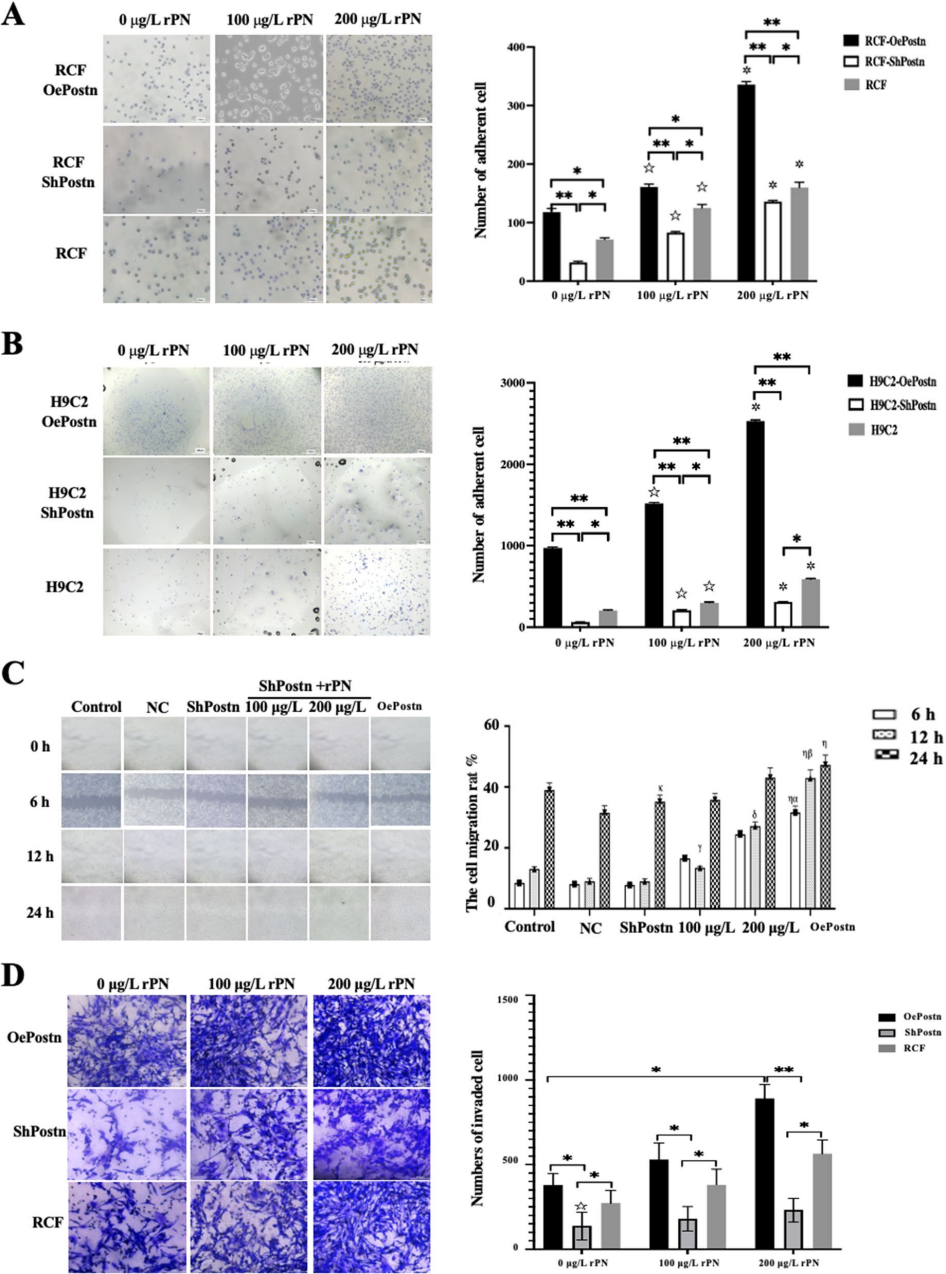
POSTN positively regulates cells adhesion, migration and invasion. ShPostn, knocking down *Postn*; OePostn, overexpressesing *Postn*; rPN, recombinant POSTN protein. **A** and **B** plots were from RCF and H9c2 cell adhesion experiments, respectively. Among them, ☆ Compared with 0 μg/L rPN group, the number of adhered cells in the 100 μg/L rPN group was increased significantly,* P* < 0.05. ^✲^ Compared to the 0 μg/L rPN group, the number of adherent cells in the OePostn group was significantly higher than that in the 200 μg/L rPN group. Fig **C** is the results of RCF cell migration assay. ^α^ After 6 h, the migration ability of OePostn group was significantly higher than that of the control, NC, ShPostn and rPN group, *P* < 0.05; ^β^after 12 h, the migration ability of overexpression group was significantly higher than that of control, NC, ShPostn and rPN group, *P* < 0.05; ^γ^ after 12 h, the migration ability of ShPostn + 100 μg/L rPN was significantly higher than that of ShPostn and NC group, ^P^ < 0.05; ^δ^after 12 h, the migration ability of ShPostn + 200 μg/L rPN was significantly higher than that of control, ShPostn and ShPostn + 100 μg/L rPN group, *P* < 0.05; ^κ^after 24 h, the migration ability of ShPostn group was significantly lower than that of other groups, *P* < 0.05. As shown in Fig. **D**, which is Transwell's experiment,☆ indicated that there was significant difference between 0 μg/L rPN and 200 μg/L rPN in ShPostn group. *P < 0.05, **P < 0.001

The components of the ECM can influence cellular biological activities. We investigated alterations in cellular adhesion, migration, and invasion abilities following *Postn* knockdown and overexpression. As depicted in Fig. 6, knockdown of *Postn* resulted in a significant decrease in cell adhesion, migration, and invasion abilities, compared to the control (*P* < 0.05). Conversely, overexpression of *Postn* led to a significant increase in cell adhesion, migration, and invasion abilities (*P* < 0.05). Moreover, exogenous rPN protein dose-dependently enhanced the adhesion number of RCF and H9c2 cells (*P* < 0.05), and rPN addition reversed the negative effect on cell migration after *Postn* knockdown in a dose-dependent manner. These findings indicate that POSTN positively regulates cell adhesion, migration, and invasion.

### *Postn* knockdown reduces differentiation of P19 cells into cardiomyocytes

**Fig. 7:**
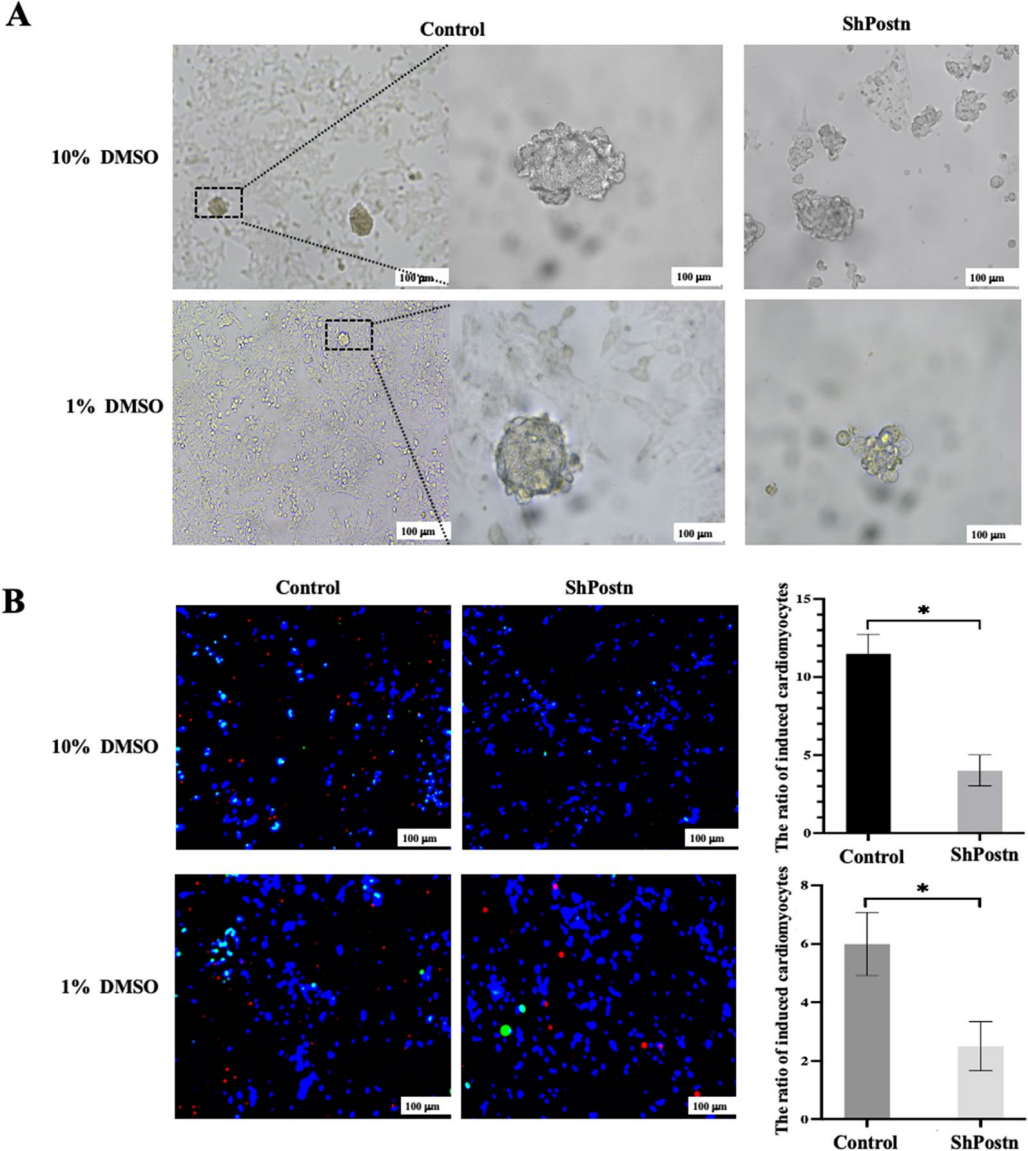
*Postn* knockdown reduces differentiation of P19 cells into cardiomyocytes. ShPostn, knocking down *Postn*; DMSO, Dimethyl sulfoxide; **A** shows the cells induced with 1% DMSO exhibited better morphology than those induced with 10%. **B** shows the cells at day 15 of induction by DMSO, where both anti-a-SAM (green) and anti-cTnT positive (red) are considered successfully induced DAPI, blue represent nucleus. These findings suggest that knock-down of Postn reduces the degree of differentiation of P19 cells into cardiomyocytes. *P < 0.05

It is currently known that P19 cells can be induced to differentiate into cardiomyocytes under DMSO induction. We aimed to to assess the impact of *Postn* knock-down in P19 cells via lentiviral transduction on their ability to differentiate into cardiomyocytes following DMSO induction. Successful induction was defined as the presence of positive staining for both anti-α-SAM and anti-cTnT in P19 cells 15 days after DMSO induction. As depicted in Fig. 7A, the cells induced with 1% DMSO exhibited better morphology than those induced with 10%. As illustrated in Figure B, the ability of P19 cells with *Postn* knock-down to differentiate into cardiomyocytes was significantly reduced, regardless of whether they were induced with 10% or 1% DMSO. (*P* < 0.05).

### The TGFβ-Smad2/3 signaling pathway modulates the expression of POSTN

**Fig. 8:**
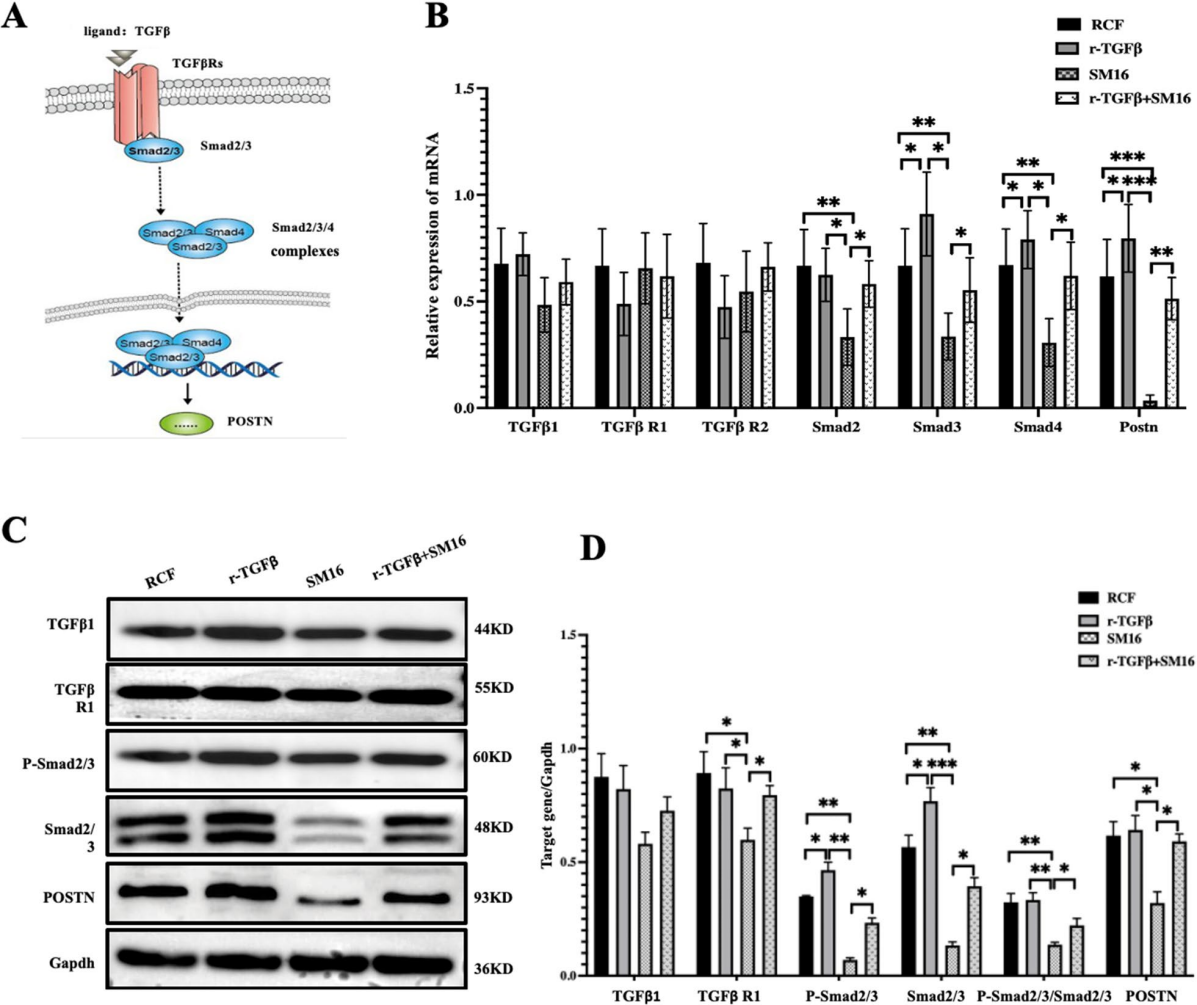
The expression of PSTN regulated by TGFβ-Smad2/3 signaling pathway. r-TGFβ, TGFβ recombinant protein, and SM16 are agonists and inhibitors of the TGFβ-Smad2/3 signaling pathway, respectively. **A** depicts a schematic of the TGFβ- Smad2/3 signaling pathway's regulatory effect on POSTN expression. **B** showed that the expression of TGFβ downstream genes *Smad2* mRNA, *Smad3* mRNA, *Smad4* mRNA and *Postn* mRNA were significantly decreased in the SM16 group (*P* < 0.05), and the expression trend was opposite in the r-TGFβ group (*P* < 0.05), while there was no significant difference between the r-TGFβ combined with SM16 group and the control group. The results in Fig **C** and **D** suggest that these TGFβ downstream protein expression trends are similar to the results of qPCR. *P < 0.05, **P < 0.001

Our research findings above have demonstrated that changes in POSTN expression levels can alter cell behaviors such as adhesion, migration, and invasion. However, the underlying signaling pathway through which POSTN exerts its effects remains unknown. POSTN is an ECM protein, and the TGFβ-Smad2/3 signaling pathway is a crucial ECM-related signaling pathway that has known to play a significant role in heart development. Therefore, we investigated whether POSTN participates in CHD development through the TGFβ-Smad2/3 signaling pathway. As depicted in Fig. 8, compared to the control group, the SM16 group (TGFβ-Smad2/3 signaling pathway inhibitor) exhibited a significant decrease in *Smad2* mRNA, *Smad3* mRNA, *Smad4* mRNA, *Postn* mRNA, and protein expression of TGFβR1, Smad2-3, P-Smad2-3, and POSTN (*P* < 0.05), while the opposite trend was observed in the TGFβ group (TGFβ-Smad2/3 signaling pathway activator) (*P* < 0.05). However, these increasing and decreasing trends were offset by the simultaneous addition of TGFβ and SM16. These results indicate that POSTN expression levels can be modulated through the TGFβ-Smad2/3 signaling pathway.

### Logistic regression analysis and decision curve analysis

**Fig. 9:**
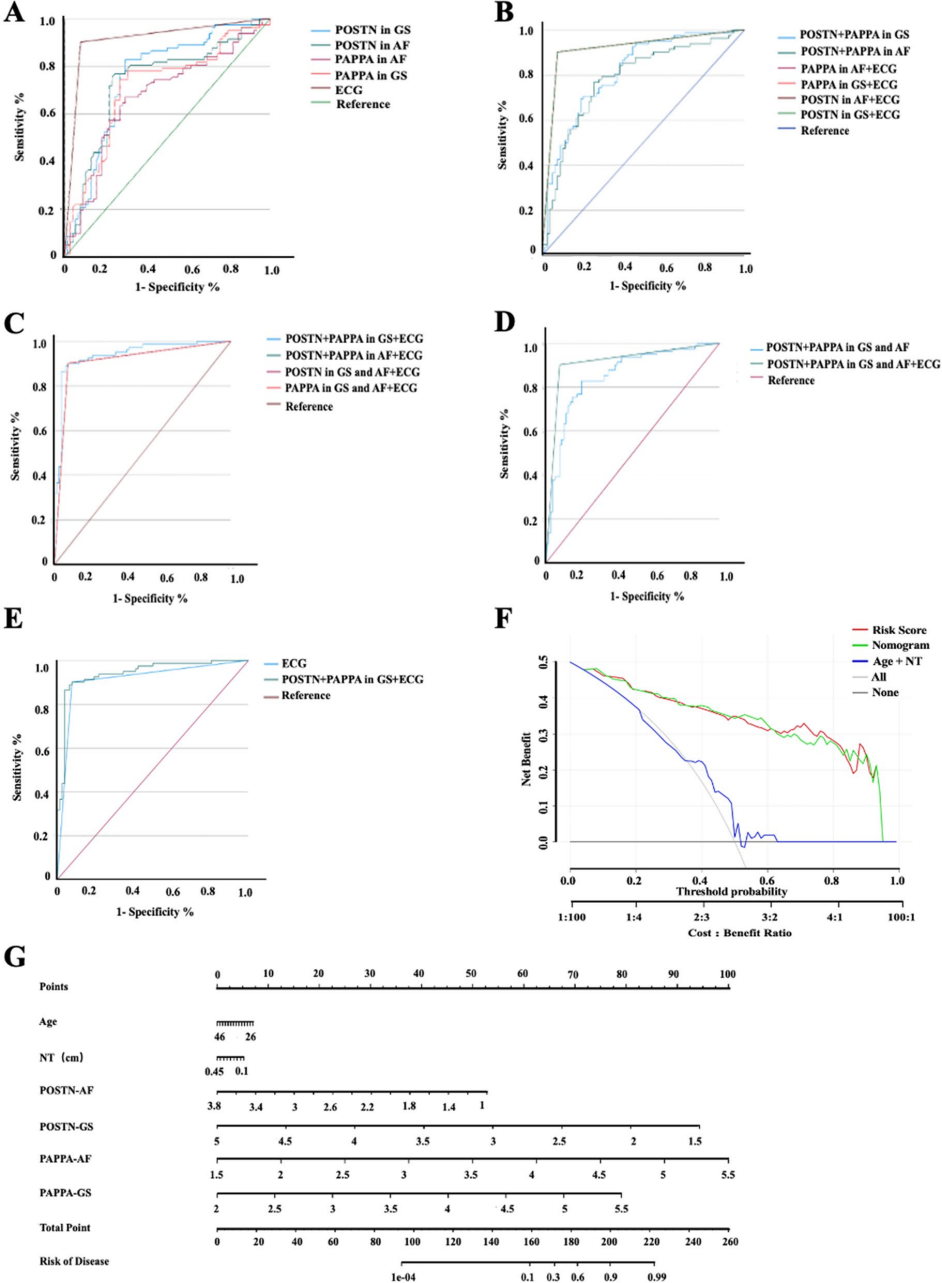
Logistic regression analysis and decision curve analysis for diagnosis of CHD. GS, gravida serum; AF, amniotic fluid; ECG, echocardiography; NT, fetal nuchal translucency. **A**-**E** depict the logistic regression analyses. **A** displays that ECG showed the best diagnostic effect for fetal CHD among the single index. **B** indicate the diagnostic effects of the double-index is better than that of the single index, when ECG is excluded from the analysis. **C** illustrates that POSTN and PAPPA in GS among the best diagnostic effect for fetal CHD among the three indexes. **D** presents the regression analysis with multiple variables (more than 3). **E** shows ROC of POSTN and PAPPA in GS combined with ECG and ECG alone**.** In addition, we have listed all parameters such as AUC, sensitivity, specificity, etc. for each group in the Logistic regression analysis in Additional file 3. **F** is the decision curve analysis (DCA) curve, which shows that the predictive ability of the comprehensive model group(include POSTN, PAPPA and the two clinical indicators, namely NT and age) is significantly higher than that of the diagnosis group without using above CHD markers and clinical indicators. **G** is the nomograms of the diagnostic model, which represents the prediction of disease scores using a comprehensive model combining clinical indicators and gene expression

As previously mentioned, fetal echocardiography is currently the primary method for diagnosing CHD in fetuses, but it has a certain rate of missed diagnosis. However, the combination of multiple indicators can improve the diagnostic rate of the disease. Therefore, we explored the diagnostic value of multiple indicators in the diagnosis of fetal CHD. As depicted in Fig. 9A, ECG showed the highest diagnostic effect for fetal CHD among the single indices of POSTN, PAPPA, or ECG. We have presented AUC, sensitivities, specificities and all other parameters of the diagnostic model in Additional file 3. Among the double-index diagnostic models, the AUC values for diagnosing fetal CHD using serum POSTN combined with serum PAPPA, amniotic fluid POSTN combined with PAPPA, ECG combined with POSTN or PAPPA were 0.85, 0.821, and 0.913, respectively, with sensitivities, specificities, negative predictive values(NPV), and positive predictive values(PPV) ranging between 70 and 90%. From the perspective of noninvasive prenatal diagnosis/screen, we focused on POSTN and PAPPA in GS combined with ECG, which showed significantly higher AUC values (0.942 vs 0.913), specificity (0.962 vs 0.91), and NPV (0.923 vs 0.900) compared to ECG alone (Fig. 9C). These results suggest that the diagnostic effects of the double-index of POSTN and PAPPA (Fig. 9B) is higher than that of the single index (Fig. 9A), when ECG is excluded from the analysis. Importantly, POSTN and PAPPA in GS combined with ECG can further improve the diagnostic rate of fetal CHD. Therefore, POSTN and PAPPA possess certain clinical value for diagnosing/screen fetal CHD.

## Discussion

This study provides the first evidence that *Postn* knockout in C57BL/6 fetal mouse can lead to CHD, including atrial and ventricular septal defects and aortic arch malformations. Additionally, we also discovered that the expression level of POSTN can be modulated by TGFβ-Smad2/3 signaling pathway and result in affecting the biological behavior of cardiac cells, thereby participating in the development of fetal mice cardiac malformations. Besides, POSTN and PAPPA in GS combined with ECG can further improve the diagnostic rate of fetal CHD. Previous literature reviews support the potential pathological and physiological role of POSTN in cardiovascular disease. However, due to the lack of clinical data, further research is needed to explore its potential as a new cardiac biomarker [12]. Nonetheless, our findings not only further support the potential of POSTN as a new cardiac disease biomarker but also validate the reliability of our previous proteomic results [10].

Although there have been no prior studies on POSTN in fetal diseases or its expression in GS and AF, many studies have reported differential expression of POSTN (usually up-regulated) in heart valvulogenes [13], myocardial fibrosis [14], coronary artery disease [15], acute myocardial injury [16], allergic disease, inflammation, and cancer [17]. In line with prior research [18], our study emphasizes the significance of POSTN, a non-structural ECM protein, in influencing cellular behavior, and regulating ECM homeostasis. Increasing evidence suggests that ECM plays a significant role in cells communication, function, and differentiation during embryonic heart development [19].

Markwald et al. demonstrated that *Postn* knockout mice leads to abnormal development of heart valves [20], because POSTN is necessary for maturation of non-myocardial cardiac cell lineages and ECM stability in the heart [21]. Our results suggest that decreased collagen and disrupted structure in cardiac ECM both in *Postn*^*−/−*^ and *Postn*^±^ mouse, as well as increased chromatin and abnormal mitochondrial proliferation in *Postn*^*−/−*^ mouse cardiac nuclei. It is known that heterochromatin cannot be transcribed or translated, but can regulate euchromatin in a reverse manner; mitochondrial proliferation is an adaptive response to chronic non-specific cell damage. The augmentation in heterochromatin and mitochondrial proliferation is a coordinated process. Abnormalities in the components of the ECM, such as collagen, can disrupt the internal homeostasis of the ECM, thereby impeding the normal transmission of signaling pathways within and outside the cell. After receiving erroneous signals, cells undergo mitochondrial proliferation (which increases the energy supply and exerts negative regulation on chromatin) and exhibit abnormal organelle morphology. Therefore, the functions of the heart ECM and its sub-organelles are closely coordinated and play an important role in heart development.

Although limited research has been conducted on the association between PAPPA and cardiac malformations, PAPPA is known to be associated with adverse outcomes in many obstetric diseases. Specifically, decreased PAPPA levels in the first trimester are associated with adverse pregnancy outcomes [22], while elevated serum PAPPA levels are observed in pregnant women with hypertension [23]. In addition, lower PAPPA levels and increased NT thickness in the first trimester are associated with an increased risk of CHD [24]. Our study found that PAPPA levels in GS, AF, and myocardial tissue of the CHD group were higher than those in the control group, which may be related to differences in gestational week samples across studies. It is known that PAPPA levels in maternal circulation increase steadily between weeks 7 and 40 of gestation before stabilizing or even decreasing during the last 4 weeks of gestation [25]. PAPPA, a metalloprotease secreted by the human placenta, regulates insulin-like growth factor bioavailability and is crucial for maternal–fetal glucose metabolism and fetal development.

It is widely acknowledged that an area under the curve (AUC) value closer to 1 in a receiver operating characteristic (ROC) curve indicates better diagnostic performance, with an AUC above 0.9 indicating high accuracy, and an AUC ranging from 0.7 to 0.9 indicating some degree of accuracy. However, an AUC above 0.7 is considered relatively satisfied for clinical use. The AUC value of ECG was 0.913, with a sensitivity of 90.2% and a specificity of 91%, and positive and negative predictive values of 93.6% and 90.0%, respectively (Fig. 9A and Additional file 3), which is consistent with previous literature [26]. The AUC, specificity, and negative predictive value of maternal peripheral serum POSTN and PAPPA in combination with ECG for the diagnosis of CHD were significantly higher than those of ECG alone (Fig. 9C). This study has significant clinical implications, as the combination of POSTN and PAPPA, which are objective markers of fetal CHD in GS, along with ECG, can lower the chance of missed diagnoses of fetal CHD. Furthermore, markedly abnormal expression of POSTN and PAPPA in GS can be identified before the detection of fetal CHD by ECG, serving as an early warning sign and facilitating the triage of these CHD fetuses to hospitals with superior medical resources.

Compared to previous studies, this study has implemented several enhancements. We did not limit our investigation to the proteomic screening level, but instead conducted cell and animal experiments to further validate the potential CHD biomarker POSTN gene in mice with cardiac malformations after the initial proteomic screening. We also explored the pathogenesis of POSTN in CHD and further investigated the non-invasive diagnostic value of POSTN and PAPPA in fetal CHD. We not only focus on the mechanisms of action of CHD biomarkers but also place great emphasis on the translational value of these biomarkers for clinical applications. However, the study has certain limitations. We did not observe significant differences in cardiac function between homozygous, heterozygous, and wild-type mice, which may be attributed to the sample size. Due to the relatively low incidence of CHD compared to common infections, the sample size of CHD cases collected within a short period of time is limited. Therefore, in the future, we will strive to obtain clinical samples from multiple centers with large datasets to validate the clinical value of POSTN and PAPPA in the screening of fetal CHD.

## Conclusions

Our study uncovers, for the first time, that fetal mice with *Postn* knockout exhibit cardiac structural malformations, myocardial ECM disorders, and cardiomyocyte suborgan abnormalities. POSTN may participate in the occurrence of fetal CHD by regulating cellular behaviors such as proliferation and adhesion via the TGFβ-Smad2/3 signaling pathway, and influencing the cardiac ECM. Importantly, POSTN and PAPPA may serve as noninvasive prenatal diagnostic biomarkers for fetal CHD and have clinical applications. This study is poised to establish a theoretical framework that will pave the way for mitigating the incidence of birth defects, while simultaneously advancing a novel and non-invasive approach for prenatal screening of fetal CHD.

### Supplementary Information

**Additional file 1: **Mouse gene identification and cell knockdown and overexpression of POSTN.

**Additional file 2: **Specific steps for adhesion, migration, invasion, and differentiation experiments.

**Additional file 3: **All parameters for each group in the Logistic regression analysis.

## Data Availability

The datasets used in this study are available upon reasonable request from the corresponding author.

## Abbreviations

AF: Amniotic fluid
ASD: Atrial septal defect
AUC: Area under curve
CHD: Congenital heart disease
DMSO: Dimethylsulfoxide
ECG: Echocardiography
ECM: Extracellular matrix
ELISA: Enzyme linked immunoso
GS: Gravida serum
H9C2: Cardiomyocytes 2
HE: Hematoxylin–eosin staining
INS: Interventricular septum
LA: Left atrium
LV: Left ventricular
NIPT: Non-invasive prenatal testing
NPV: Negative predictive value
NT: Nuchal translucency
PAPPA: Pregnancy-associated plasma protein
POSTN: Periostin
PPV: Positive predictive value
RA: Right atrium
RCF: Rat cardiac fibroblasts
ROC: Receiver operating characteristic curve
RT-qPCR: Real-time quantitative polymerase chain reaction
RV: Right ventricular
TGF-β: Transforming growth factor beta
VSD: Ventricular septal defect
