# Supplementary material for: The comprehensive study on the role of POSTN in fetal congenital heart disease and clinical applications
Source: J Transl Med. 2023 Dec 11;21:901. doi: 10.1186/s12967-023-04529-1 (PMC10714640; doi:10.1186/s12967-023-04529-1)
Supplement: Supplementary file 1 — Additional file 1: Mouse gene identification and cell knockdown and overexpression of POSTN. [file 12967_2023_4529_MOESM1_ESM.docx]

**Appendix 1**

**1、Mouse genotype identification**

**Table 1-1 Primer sequences**

| **Genes** | **Sense (5’-3’)** | **Antisense (5’-3’)** |
| --- | --- | --- |
| Primers1 | AATGTGATTGCCCCTAATTTTGCC | TCATAGTTGCACACACTAAGGCA |
| Primers2 | TAGATTGCTGTTCCAAGATTGTCAG | TCATAGTTGCACACACTAAGGCA |
| Primers3 | AATGTGATTGCCCCTAATTTTGCC | CCATGCCTACATTGAACAAAACAC |

**Table 1-2 PCR reaction system (25 μL)**

| Components | Volume（μL） |
| --- | --- |
| ddH_2_O | 9 |
| Primer sense | 1 |
| Primer antisense | 1 |
| Premix Tap | 12.5 |
| DNA | 1.5 |

**Table 1-3、PCR reaction conditions.**

| Steps | Temperature | Duration | Cycles |
| --- | --- | --- | --- |
| Pre-denaturation | 94°C | 3min |  |
| Denaturation | 94°C | 30S | 35 |
| Annealing | 60°C | 35S |  |
| Stretching | 72°C | 35S |  |
| Stretching again | 72°C | 5min |  |

The above primers were synthesized by Qingke Biotech Co., Ltd. The 0.5×TBE (TSG002, Qingke Biotech) solution was prepared with a 2% agarose solution (TSJ001, Qingke Biotech) and the agarose gel was prepared by boiling the 2% agarose solution in a microwave twice and then adding nucleic acid dye (TSJ002, Qingke Biotech) before pouring into the gel casting tray. After cooling, electrophoresis was performed by loading 7 μL of PCR product and 5 μL of DNA ladder (RM19007, ABclonal, China) into each well. The electrophoresis buffer was 0.5×TBE, and the electrophoresis conditions were 300 V for 20 min. The homozygous: one band of 427 bp; The heterozygous: three bands at 427 bp, 440 bp, and 412 bp; The wild-type: two bands at 440 bp and 412 bp.

**二、RT-qPCR**

**Table 2-1 Reaction system (16 μL) for the removal of genomic DNA.**

| **Reagent** | **volume（μL）** |
| --- | --- |
| 4 × gDNA wiper Mix | 4.0 |
| Total RNA | 2.0 |
| RNase Free dH_2_O | 10.0 |

**Table 2-2 Reaction conditions for genomic DNA removal (16 μL)**

| **Temperature** | **Time** |
| --- | --- |
| 42°C | 2min |
| 4°C | - |

**Table 2-3 Reverse transcription system (20 μL)**

| **Reagent** | | **Volume（μL）** |
| --- | --- | --- |
| Reaction components for genomic DNA removal. | | 16.0 |
| HiScript II qRT SuperMix II^a^ | 4.0 | |

**Table 2-4 Reverse transcription conditions（20 μL）**

| **Temperature** | **Time** |
| --- | --- |
| 58°C | 15min |
| 85°C | 5s |
| 4°C | - |

**Table 2-5 Primers used for RT-qPCR and sequences**

| **Genes** | **Sense (5’-3’)** | **Antisense (5’-3’)** |
| --- | --- | --- |
| PAPPA | AGAGGAGGAGTTGGCAGGAG | CGACTTGATTGGGCGTGA |
| POSTN | GATGTGACGGTGACAGTAT | GTTTGGCAGAATCAGGAA |
| GAPDH | AGGTCCACCACTGACACGTT | GCCTCAAGATCATCAGCAAT |

**Table 2-6 Composition of the RT-qPCR system (25 μL)**

| **Reagent** | **Volume（μL）** |
| --- | --- |
| 2 x Taq Master | 10 |
| Sense | 0.4 |
| Antisense | 0.4 |
| DNA template | 2.0 |
| dH_2_O | 7.2 |

**Table 2-7 RT-qPCR reaction conditions (25 μL)**

|  | **Temperature** | **Time** |
| --- | --- | --- |
|  | 95°C | 3min |
| Cycle 335 | 95°C | 15s |
|  | 60°C | 15s |
|  | 72°C | 30s |
|  | 72°C | 5min |

**表2-8 RT-qPCR使用的引物及其序列**

| Genes | **Sense (5’-3’)** | **Antisense (5’-3’)** |
| --- | --- | --- |
| m-POSTN | CCACATCCTAAATACCCTC | ACCATCTTGATTCCGTTA |
| m-GAPDH | TCAACGGCACAGTCAAGG | TTAGTGGGGTCTCGCTCC |
| r-TGFbeta1 | CCCACTGATACGCCTGAG | TGAAGCGAAAGCCCTGTA |
| r-TGFbetaR1 | TCGCCCTTCATTTTCAGA | TTTGCCGATGCTTTCTTG |
| r-TGFbetaR2 | CGCCAACAACATCAATCA | CAGCCACGGTCTCAAACT |
| r-Smad2 | GTGTCTCATCGGAAAGGG | CTCTGGTAGTGGTAAGGGT |
| r-Smad3 | TAGCGACCACCAGATGAAC | TCGTAGTAGGAGATGGAGCA |
| r-Smad4 | TGGATTCACTGCTCAGCCAG | CGGGTAGATCTTGTGGACGG |
| r-POSTN | TCTCTGAAGCCGGATGGAGA | ATGTTGGTGACTCCGGGTTC |
| r-GAPDH | CTCCCATTCTTCCACCTTTG | TGGTCCAGGGTTTCTTACT |

Notes: m, mouse; r, rat.

**三、 Western Blot, WB**

Anti POSTN (ab219057, abcam, USA),

Anti PAPPA (ab174314, abcam, USA),

Anti SMAD2/SMAD3 (GT111123, GeneTex, USA),

Anti Phospho Smad2+Smad23 (AP0548, ABclonal, China),

Anti TGFbeta1 (21898-1-AP, Sanying, China),

Anti a-SMA (BM0002, BOSTER, China),

Anti TGFbetaR1 (A16983, ABclonal, China),

Anti TNN2 (abs135781, sin China)

GAPDH (60004-1-Ig, Sanying, China),

Goat anti rabbit IgG (abs20040ss, absin, China),

Goat anti mouse IgG (SA00001-1, Sanying, China).

**四、Reagents for cell culture, knockdown, and overexpression of *Postn***

The complete culture media for H9c2, RCF, and P19 cells include: DMEM (without 110 mg/L sodium pyruvate, Biosharp, Anhui, China), DMEM (with 110 mg/L sodium pyruvate, Procell, Wuhan), and MEM-α (Procell, Wuhan), all of which contain 10% fetal bovine serum (Sijiqing, Hangzhou) and 1% penicillin/streptomycin (C0222, Biyuntian, Shanghai). Targeted knockdown of Postn in RCF and H9c2 cells was achieved using siRNA. The cells were cultured in a CO2 incubator (MCO-170MUVL, Panasonic, Japan). Lipofectamine 3000 reagent kit (Invitrogen, USA) was used.

**Table 4: shRNA sequences.**

| **Genes** | **Sense (5’-3’)** | | **Antisense (5’-3’)** |
| --- | --- | --- | --- |
| shPOSTN(rat) | | GGAGACAAAGUGGCUUCUGAATT | UUCAGAAGCCACUUUGUCUCCTT |
| shPOSTN(mouse) | | GAUGCCUAUUGACCAUGUUUATT | UAAACAUGGUCAAUAGGCAUCTT |
