# Supplementary material for: The comprehensive study on the role of POSTN in fetal congenital heart disease and clinical applications
Source: J Transl Med. 2023 Dec 11;21:901. doi: 10.1186/s12967-023-04529-1 (PMC10714640; doi:10.1186/s12967-023-04529-1)
Supplement: Supplementary file 2 — Additional file 2: Specific steps for adhesion, migration, invasion, and differentiation experiments. [file 12967_2023_4529_MOESM2_ESM.docx]

**Cell Adhesion**

Cell adhesion ability can be altered due to changes in internal and external environments (extracellular matrix). The groups included the Control group (RCF/H9c2), ShPN group (Postn knockdown RCF/H9c2 cells), OePN group (Postn overexpression RCF/H9c2 cells), rPN100μg/L (recombinant POSTN protein added to RCF/H9c2 cell culture dish at 100μg/L), ShPN+rPN100μg/L group (Postn knockdown RCF/H9c2 cell culture dish with added recombinant POSTN protein at 100μg/L), OePN+rPN100μg/L group, rPN200μg/L (recombinant POSTN protein added to RCF/H9c2 cell culture dish at 200μg/L), ShPN+rPN200μg/L group, and OePN+rPN200μg/L group. The specific steps are as follows:

(1) Dilute DMEM and matrix gel in a ratio of 1:300 to prepare artificial basement membrane.

(2) Plate matrix gel (2μg/50μL) onto a 96-well plate and air-dry it in a laminar flow hood.

(3) Add an appropriate amount of serum-free DMEM to each well of the 96-well plate and incubate for 60 minutes to remove excess matrix gel.

(4) Plate different groups of cells in triplicate and incubate in a cell culture incubator for 1 hour.

(5) Remove the culture medium, wash the cells three times with PBS to remove unattached cells.

(6) Fix the cells with 4% paraformaldehyde for 15 minutes.

(7) Stain the cells with crystal violet for 15 minutes and then remove the staining solution with double-distilled water.

(8) Count the number of cells under a microscope.

**Migration assay**

Blank areas were artificially created in the culture dishes of fully grown monolayer RCF and H9c2 cells, and the cells automatically migrated towards the blank areas. The scratch width was measured at 0, 6, 12, and 24 hours after scratch, and the efficiency of cell migration was compared. The experiment was divided into six groups: Blank group (RCF), NC group (negative control group), ShPN group (RCF cells with knocked-down *Postn* expression), OePN group (RCF cells with overexpressed *Postn* expression), ShPN+rPN100ug/L group (RCF cells with knockdown *Postn* expression and added with 100 ug/L recombinant POSTN protein), and ShPN+rPN200ug/L group (RCF-ShPN cells added with 200 ug/L recombinant POSTN protein). The specific steps were as follows:
(1) Use a ruler to draw horizontal lines evenly on the back of a 6-well plate with a marker pen to locate the observation sites.
(2) Add 5×10^5^ cells to each well of each group, which can be fully covered after overnight incubation.
(3) The next day, use a yellow pipette tip of 200 μL to scratch the cells perpendicular to the horizontal line on the back of the plate.
(4) Wash the cells slowly with PBS three times, and remove the cells and debris, then add 1% FBS culture medium.
(5) Place the plate in a 37°C, 5% CO2 incubator for culture.
(6) Take pictures under a microscope at 0, 6, 12, and 24 hours.
(7) Analyze using Image pro plus: scratch width (=initial scratch area - scratch area at a certain point)/initial width, cell migration rate = (initial width - end width)/initial width.

**Transwell**

The principle of this experiment is to simulate ECM using a layer of polycarbonate membrane (with membrane pores covered by Matrigel) to separate high-nutrient and low-nutrient culture media. Typically, cells are seeded in the low-nutrient culture medium and must secrete hydrolases and undergo deformation to pass through the Matrigel-coated membrane, after which the number of cells that enter the lower chamber can reflect their invasive ability. The groups include: control group (RCF cells), ShPN group (RCF cells with knocked-down *Postn* gene expression), OePN group (RCF cells with overexpressed *Postn* gene expression), r-PN100ug/L (RCF cells with added recombinant *Postn* protein at 100ug/L), ShPN+rPN100ug/L (RCF cells with knocked-down *Postn* gene expression and added recombinant POSTN protein at 100ug/L), OePN+r-PN100ug/L group (RCF cells with overexpressed *Postn* gene expression and added recombinant POSTN protein at100ug/L), r-PN200ug/L, ShPN+rPN200ug/L, and OePN+r-PN200ug/L. The specific steps are as follows:

(1) Matrigel coating of Transwell chambers: Dilute 50mg/L Matrigel gel in culture medium at a ratio of 18, add200 μL of the diluted Matrigel gel to each Transwell chamber, and incubate the chambers in a culture incubator for 2 hours before discarding the liquid in the chambers.

(2) Cell seeding: Digest cells from different groups with trypsin, make single-cell suspensions with serum-free culture medium, and add about 1105 cells (200 μL culture medium) to each Transwell chamber. Add 200 μL culture medium containing 20% FBS to the lower chamber as a chemoattractant. Incubate the chambers in a culture incubator for 48 hours.

(3) Staining, observation, and counting: Take the chambers from 24-well plate, fix them with 4% paraformaldehyde for 20 minutes, discard the paraformaldehyde, and stain the cells with crystal violet solution (Aladdin) for 15 minutes. Wash the chambers with PBS for 3 times to remove unbound crystal violet. Use a cotton swab to wipe off excess, observe and take pictures of the chambers under an inverted microscope. Randomly select 10 fields of view (40×) from each chamber and count the cells.

**Induction of P19 cell differentiation into cardiomyocytes**

When the cell culture flask (25cm2) reaches approximately 90% confluency, aspirate the liquid, wash twice with PBS, add 1mL of 0.25% trypsin for digestion. Observe under a microscope as the cells gradually shrink and detach. Terminate digestion by adding complete culture medium and 3 mL of DMEM containing 10% FBS. Pipette up and down to form a single cell suspension at a concentration of 3.5×105/mL and seed into a 10 cm culture dish (pre-coated with 0.5% agarose) for differentiation induction beginning on day 1. Cells are suspended in complete medium containing 1% and 10% DMSO (10 mL) and incubated overnight in the culture incubator. On day 3, replace the equivalent amount of medium in the culture dish with 7 mL of complete medium containing DMSO. From day 3 to 5, embryoid bodies or aggregates can be observed in the culture dish. Collect the healthy aggregates using a pipette and transfer them to a 6-well plate for further culture in complete medium (without DMSO) to promote adherence to the plate. Change the medium every other day. On day 10, beating cardiomyocyte-like cells can be observed. On days 0, 4, and 10, observe the changes in cell morphology under an inverted phase contrast microscope, and collect cells. On day 15, the percentage of successfully induced differentiated cells (both a-SMA and TNN2 positive) is determined by immunofluorescence detection. The immunofluorescence steps are the same as before. The antibodies used in this experiment include anti-a-SMA (BM0002, BOSTER, China), FITC-conjugated Affinipure goat anti-Mouse IgG (H+L) (SA00003-1, Sanying), anti-TNN2 antibody (abs135781, Absin, China), and CoraLite594-conjugated Goat anti-Rabbit (H+L) (SA00013-4, Sanying).
