# Supplementary material for: The comprehensive study on the role of POSTN in fetal congenital heart disease and clinical applications
Source: J Transl Med. 2023 Dec 11;21:901. doi: 10.1186/s12967-023-04529-1 (PMC10714640; doi:10.1186/s12967-023-04529-1)
Supplement: Supplementary file 3 — Additional file 3: All parameters for each group in the Logistic regression analysis. [file 12967_2023_4529_MOESM3_ESM.docx]

**Table 2.** **All parameters for each group in the Logistic regression analysis**

| **Group** | **AUC** | ***P*** | **95%CI** | **Sensitivity** | **Specificity** | **Cut-off** | **PPV** | **NPV** |
| --- | --- | --- | --- | --- | --- | --- | --- | --- |
| ECG | 0.913 | 0.001 | 0.863，0.964 | 0.902 | 0.91 | 0.812 | 0.936 | 0.900 |
| POSTN in AF | 0.735 | 0.000 | 0.654，0.815 | 0.838 | 0.812 | 0.65 | 0.768 | 0.731 |
| POSTN in GS | 0.758 | 0.000 | 0.679，0.833 | 0.863 | 0.725 | 0.588 | 0.780 | 0.731 |
| PAPPA in AF | 0.673 | 0.000 | 0.588，0.758 | 0.713 | 0.775 | 0.488 | 0.793 | 0.731 |
| PAPPA in GS | 0.711 | 0.000 | 0.629，0.793 | 0.813 | 0.213 | 0.6 | 0.817 | 0.744 |
| POSTN+PAPPA in GS | 0.85 | 0.000 | 0.791，0.909 | 0.695 | 0.818 | 0.503 | 0.734 | 0.579 |
| POSTN+PAPPA in AF | 0.821 | 0.000 | 0.754，0.888 | 0.768 | 0.744 | 0.512 | 0.756 | 0.613 |
| POSTN in AF +ECG | 0.913 | 0.000 | 0.863，0.964 | 0.902 | 0.923 | 0.825 | 0.902 | 0.923 |
| POSTN in GS +ECG | 0.913 | 0.000 | 0.863，0.965 | 0.902 | 0.923 | 0.825 | 0.902 | 0.923 |
| PAPPA in AF +ECG | 0.913 | 0.000 | 0.863，0.966 | 0.902 | 0.923 | 0.825 | 0.902 | 0.923 |
| PAPPA in GS +ECG | 0.913 | 0.000 | 0.863，0.967 | 0.902 | 0.923 | 0.825 | 0.902 | 0.923 |
| POSTN in AF and GS +ECG | 0.913 | 0.000 | 0.863，0.964 | 0.902 | 0.923 | 0.825 | 0.902 | 0.923 |
| PAPPA in AF and GS +ECG | 0.913 | 0.000 | 0.863，0.965 | 0.902 | 0.923 | 0.825 | 0.902 | 0.923 |
| POSTN +PAPPA in AF +ECG | 0.913 | 0.000 | 0.863，0.966 | 0.902 | 0.923 | 0.825 | 0.902 | 0.923 |
| POSTN+PAPPA in GS +ECG | 0.942 | 0.000 | 0.904，0.979 | 0.866 | 0.962 | 0.828 | 0.902 | 0.923 |
| POSTN+PAPPA in AF and GS | 0.852 | 0.000 | 0.792，0.913 | 0.829 | 0.795 | 0.642 | 0.829 | 0.744 |
| ECG | 0.913 | 0.001 | 0.863，0.964 | 0.902 | 0.91 | 0.812 | 0.936 | 0.900 |
| POSTN in AF | 0.735 | 0.000 | 0.654，0.815 | 0.838 | 0.812 | 0.65 | 0.768 | 0.731 |

**Notes**：GS，gravida serum；AF，amniotic fluid；ECG，echocardiography；PPV：positive predictive value，NPV：negative predictive value。 **P* < 0.05，***P* < 0.001.
